# Arsenic Inhibits Myogenic Differentiation and Muscle Regeneration

**DOI:** 10.1289/ehp.0901525

**Published:** 2010-03-18

**Authors:** Yuan-Peng Yen, Keh-Sung Tsai, Ya-Wen Chen, Chun-Fa Huang, Rong-Sen Yang, Shing-Hwa Liu

**Affiliations:** 1 Institute of Toxicology and; 2 Department of Laboratory Medicine, College of Medicine, National Taiwan University, Taipei, Taiwan; 3 Department of Physiology and; 4 Graduate Institute of Basic Medical Science, China Medical University, Taichung, Taiwan; 5 Graduate Institute of Chinese Medical Science, School of Chinese Medicine, China Medical University, Taichung, Taiwan; 6 Department of Orthopaedics and; 7 Department of Urology, College of Medicine and Hospital, National Taiwan University, Taipei, Taiwan

**Keywords:** Akt signaling, arsenic trioxide, myogenic differentiation

## Abstract

**Background:**

The incidence of low birth weights is increased in offspring of women who are exposed to high concentrations of arsenic in drinking water compared with other women. We hypothesized that effects of arsenic on birth weight may be related to effects on myogenic differentiation.

**Objective:**

We investigated the effects of arsenic trioxide (As_2_O_3_) on the myogenic differentiation of myoblasts *in vitro* and muscle regeneration *in vivo*.

**Methods:**

C2C12 myoblasts and primary mouse and human myoblasts were cultured in differentiation media with or without As_2_O_3_ (0.1–0.5 μM) for 4 days. Myogenic differentiation was assessed by myogenin and myosin heavy chain expression and multinucleated myotube formation *in vitro*; skeletal muscle regeneration was tested using an *in vivo* mouse model with experimental glycerol myopathy.

**Results:**

A submicromolar concentration of As_2_O_3_ dose-dependently inhibited myogenic differentiation without apparent effects on cell viability. As_2_O_3_ significantly and dose-dependently decreased phosphorylation of Akt and p70s6k proteins during myogenic differentiation. As_2_O_3_-induced inhibition in myotube formation and muscle-specific protein expression was reversed by transfection with the constitutively active form of *Akt*. Sections of soleus muscles stained with hematoxylin and eosin showed typical changes of injury and regeneration after local glycerol injection in mice. Regeneration of glycerol-injured soleus muscles, myogenin expression, and Akt phosphorylation were suppressed in muscles isolated from As_2_O_3_-treated mice compared with untreated mice.

**Conclusion:**

Our results suggest that As_2_O_3_ inhibits myogenic differentiation by inhibiting Akt-regulated signaling.

Inorganic arsenic is a recognized toxicant and carcinogen. Exposure to inorganic arsenic usually incurs obvious developmental and reproductive toxicity (Willhite and Ferm 1984). People drinking arsenic-polluted well water showed an increased risk of type 2 diabetes in areas with high levels of arsenic in Taiwan and Bangladesh (Chen et al. 2007; Lai et al. 1994; Navas-Acien et al. 2006; Rahman et al. 1998). Chakraborti et al. (2003) found an increased incidence of miscarriages, stillbirths, preterm births, and low birth weights in offspring of women who drank water containing arsenic concentrations ranging from 463 to 1,025 μg/L. Alterations in muscle fiber composition and size may contribute to the development of type 2 diabetes in individuals with low birth weight (Jensen et al. 2007). Further study has shown that low-birth-weight piglets differentiate low numbers of muscle fibers during prenatal myogenesis, and affected piglets do not exhibit postnatal “catch-up” growth (Rehfeldt and Kuhn 2006). Additionally, maternal undernutrition before fetal muscle differentiation decreases the myoblast proliferation coupled with an earlier onset of differentiation to fibers, alters development of muscle fibers, and reduces the birth weight of newborn lambs (Brameld et al. 2000; Fahey et al. 2005). Moreover, Ronco et al. (2009) recently reported that exposure to cadmium during pregnancy reduces birth weight and elevates both maternal and fetal glucocorticoid levels. Such embryonic exposure to increased glucocorticoids might be related to low birth weight and later deleterious consequences in adult life (McTernan et al. 2001; Seckl and Holmes 2007). Perinatal high-dose dexamethasone exposure could also alter development of skeletal muscles in rats (Steiss et al. 1989). Therefore, we hypothesize that alteration of skeletal myogenic differentiation may be involved in low birth weight after exposure to arsenic and that arsenic may also alter muscle regeneration.

Arsenic trioxide (As_2_O_3_) induces differentiation of promyelocytic leukemia cells (Chen et al. 1997) and suppresses the differentiation of human keratinocytes (Kachinskas et al. 1994, 1997) and murine adipocytes (Trouba et al. 2000). A recent report indicated that arsenic exposure alters the expression or activity of different genes and proteins expressed in muscle and fat tissues (Diaz-Villasenor et al. 2007). However, the precise mechanism of action of arsenic on skeletal myogenic differentiation remains unknown.

Myogenesis is a process that involves three major steps: withdrawal of myoblasts from the cell cycle, subsequent expression of myotube-specific genes, and formation of multinucleated myotubes (Weintraub 1993; Weintraub et al. 1991). Myogenesis is largely regulated by the myogenic basic helix-loop-helix family of transcription factors (MyoD, myogenin, myf5, and MRF4) and myocyte enhancer factor 2, which regulate the expression of many muscle-specific genes, such as the myosin heavy chain (MHC) and creatine kinase (Lassar et al. 1994; Olson et al. 1995). Cell division is prevented during muscle differentiation by the induction of cyclin-dependent protein kinase inhibitors (Franklin and Xiong 1996; Halevy et al. 1995). Furthermore, the absence of myogenin reduces MHC expression and myotube formation, indicating that myogenin has a unique function in the transition from a determined myoblast to a fully differentiated myotube (Davie et al. 2007; Myer et al. 2001). Moreover, phosphatidylinositol 3-kinase (PI3K)/Akt (protein kinase B)–related signaling is thought to have a key role in the control of muscle gene expression during myogenic differentiation (Kaliman et al. 1996; Tamir and Bengal 2000). Altered PI3K/Akt signaling is also found in the skeletal muscle of young men with low birth weight (Jensen et al. 2008). Based on these cumulative results, in our present study we investigated the mechanism of action of As_2_O_3_ on the morphologic and biochemical differentiation of myoblastic cells *in vitro* and in an *in vivo* mouse model with skeletal muscle regeneration after glycerol injury. The results showed that As_2_O_3_ is capable of inhibiting skeletal myogenic differentiation by inhibiting Akt-regulated signaling.

## Materials and Methods

### Cell cultures

#### C2C12 mouse myoblasts

Cells were obtained from American Type Culture Collection (Manassas, VA, USA) and cultured in growth medium consisting of Dulbecco’s modified Eagle’s medium (DMEM) supplemented with 10% fetal bovine serum (FBS) and antibiotics (100 IU/mL penicillin, 100 μg/mL streptomycin) in 5% carbon dioxide (CO_2_) at 37°C.

### Primary myoblasts

Murine skeletal muscle tissues were obtained from the forelimb and hind limb of neonatal mice (2–5 days of age). Skeletal muscle biopsies (~ 0.2 g) were obtained from a 60-year-old female patient during orthopedic surgery at the National Taiwan University Hospital, with approval from the institutional ethical committee and informed consent from the patient. Different populations of muscle-derived cells have been isolated based on their adhesion characteristics and proliferation behaviors by Qu-Petersen et al. (2002). Here, the cells of an early preplate population, which possesses satellite cell characteristics (Qu-Petersen et al. 2002), were isolated and cultured for myogenic cell differentiation as described by Qu-Petersen et al. (2002). In brief, the muscles cleaned from the surrounding connective tissue were minced with sharp scissors to obtain fragments approximately 1 mm in diameter. Enzymatic digestion was carried out in Ham’s F10 medium (Invitrogen, Carlsbad, CA, USA) containing 0.2% collagenase (type XI; Sigma-Aldrich, St. Louis, MO, USA) plus 0.03% EDTA at 37°C for 1 hr with occasional shaking, and then centrifuged at 600 × *g* for 5 min. The cells were collected and incubated in dispase (Invitrogen) solution for 45 min and then incubated in 0.1% trypsin-EDTA for 30 min. The filtrate was spun at 600 × *g* for 5 min to sediment the dissociated cells. The pellet was resuspended in growth medium [Ham’s F-10 supplemented with 20% FBS, 2.5 ng/mL basic fibroblast growth factor (R&D Systems, Minneapolis, MN, USA), and 1% penicillin-streptomycin (Invitrogen)] in 5% CO_2_ at 37°C; the suspension was then plated on collagen-coated dishes. The cells with positive desmin (myogenic marker) staining were used for identification of myogenic differentiation.

### Myogenic differentiation and As_2_O_3_ treatment

For myogenic differentiation, C2C12 myoblasts and primary myoblasts were placed in a differentiation medium consisting of an equal mixture of two serum-free media (Nutrient Mixture F-12K Ham medium and MCDB201; Invitrogen) along with 2% horse serum to induce differentiation with or without As_2_O_3_ (0.1–0.5 μM) treatment. After 4 days of treatment, myoblast differentiation was determined morphologically by analysis of multinucleated myotube formation. We analyzed cells morphologically by hematoxylin and eosin (H&E) staining; H&E stains nuclei deep purple and protein pink, giving a good indication of overall myogenic progression.

### Protein extraction and immunoblotting

Cells were washed with phosphate-buffered saline (PBS) and lysed with RIPA buffer [10 mM Tris (pH 7.4), 150 mM NaCl, 1 mM ethylene glycol tetraacetic acid, 0.1% sodium dodecyl sulfate (SDS), 1 mM NaF, 1 mM sodium orthovanadate, 1 mM phenylmethylsulfonyl fluoride (PMSF), 1 μg/mL aprotinin, and 1 μg/mL leupeptin]. In this buffer, NaF and PMSF were the phosphatase inhibitor and serine protease inhibitor, respectively. The cell suspension was left on ice for 20 min and then centrifuged at 10,000 × *g* for 20 min at 4°C. We used the supernatant for the experiments. An equal amount (40 μg) of protein was separated by 10% SDS-PAGE and electrotransferred onto polyvinylidene difluoride membrane (0.2 μm) using transfer buffer (192 mM glycine, 25 mM Tris, 20% methanol, pH 8.3) followed by blocking in TBST (Tris-buffered saline/Tween-20) buffer (20 mM Tris, 150 mM NaCl, 0.01% Tween-20, pH 7.5) supplemented with 5% nonfat powdered milk. The membranes were then probed with the primary antibodies [anti-extracellular signal-regulated kinase (ERK), anti-Akt, anti-phosphorylated Akt (Ser473), anti-myogenin, and anti-MHC; Santa Cruz Biotechnology, Santa Cruz, CA, USA] overnight at 4°C, followed by incubation with the secondary goat anti-rabbit or anti-mouse antibodies conjugated with horseradish peroxidase. The blots were developed using an enhanced chemiluminescence reagent detection system according to the manufacturer’s protocol (Millipore Corporation, Billerica, MA, USA). Densitometric analysis was carried out using Molecular Analyst software (version 1.3; BioRad, Hercules, CA, USA). The extent of phosphorylation in terms of relative band intensity was quantified by scanning densitometry using a GS-670 imaging densitometer (BioRad, Hercules, CA, USA), with an arbitrary value of 1.0 given to the respective control samples of each experiment.

### Creatine kinase activity assay

Cells were washed twice in cold PBS and lysed in lysis buffer. Lysates were centrifuged for 10 min at 13,000 × *g*, the supernatants were collected, and the protein contents in the samples were measured using bicinchoninic acid protein assay reagent (Thermo Scientific, Rockford, IL, USA). Creatine kinase activity was measured using a commercial kit (Teco Diagnostics, Anaheim, CA, USA). We calculated the activity of creatine kinase (units per milligram of protein) after correction for total protein.

### Immunocytochemistry

Cells cultured in 6- or 12-well dishes were washed with PBS followed by fixation for 10 min with 4% paraformaldehyde in PBS. Cells were then washed three times for 1 min each with PBS and permeabilized in PBS containing 0.2% Triton X-100/PBS for 5 min at room temperature. After three washes for 1 min each with PBS, cells were blocked with 5% bovine serum albumin in PBS plus 0.2% Triton X-100 for 30 min and then incubated overnight at 4°C with polyclonal rabbit anti-myogenin and anti-MHC primary antibodies diluted with blocking solution to 1:100. After four additional washes with PBS, cells were incubated for 1 hr at room temperature in the dark with goat anti-rabbit IgG1-fluorescein isothiocyanate secondary antibodies diluted in blocking solution to 1:1,000. 4′,6′-Diamino-2-phenylindole-2HCl (DAPI) nuclear dye was added with secondary antibodies at a final concentration of 400 ng/mL. Finally, cells were washed three times in PBS. Fluorescent images were captured by a Leica DMIL inverted microscope equipped with a charged-coupled device camera and SPOT software, version 4.6 (Diagnostic Instruments, Sterling Heights, MI, USA).

### Transient transfection

We performed transient transfections using a Lipofectamine 2000 reagent (Invitrogen) following the manufacturer’s instructions. Briefly, 5 × 10^5^ cells were seeded in six wells and incubated at 37°C in 5% CO_2_. After rinsing with serum-free and antibiotic-free medium, the cells were transfected separately with a control pcDNA3.1 empty vector or a constitutively active form of *Akt* [myristoylated (myr) *Akt*], which was a gift from J.L. Ko (Chung Shan Medical University, Taichung, Taiwan) and M.L. Kuo (National Taiwan University, Taipei, Taiwan) (Kuo et al. 2001), and incubated at 37°C in 5% CO_2_ for 6 hr. The medium was replaced with DMEM containing 10% FBS. We determined the efficiency of transfection (~ 40–50%) using an equal amount of a plasmid encoding the green fluorescent protein under the cytomegalovirus promoter.

### Arsenic-treated mice

Male ICR mice (20–25 g body weight) were purchased from the Animal Center of the College of Medicine, National Taiwan University (Taipei, Taiwan). The Animal Research Committee of the College of Medicine conducted the study in accordance with the *Guide for the Care and Use of Laboratory Animals* (Institute of Laboratory Animal Resources 1996). The animals were treated humanely and with regard for alleviation of suffering. Mice were housed in a room at a constant temperature of 22 ± 2°C with 12-hr light/dark cycles. Mice were exposed to As_2_O_3_ through drinking water containing 0.5 or 5 ppm (dissolved in distilled water) for 8 consecutive weeks (*n* = 16 mice/group). To prevent or minimize oxidation of As_2_O_3_, the water containing As_2_O_3_ was freshly prepared every 3–4 days.

### Experimental glycerol myopathy

Skeletal muscle injury and regeneration in mice were induced by glycerol injection according to a modification of the method described by Kawai et al. (1990). Glycerol (0.1 mL of 50%, vol/vol) was injected into the mouse soleus muscle under ketamine (30 mg/kg) and xylazine (4 mg/kg) anesthesia. Contralateral muscles served as controls for each animal. Mice were sacrificed 3, 5, or 12 days after glycerol injection, and the soleus muscles were removed. Arsenic exposure continued during glycerol injection until sample collection. We recorded body weights and the weights of skeletal muscles. Some muscles were fixed in 10% formalin and embedded in paraffin. Sections (5 μm) were cut in the middle part of the muscle belly (fleshy part of the muscle) to obtain the largest cross-section area and then stained with H&E or with Masson’s trichrome to detect collagen (fibrosis). The myofibers containing centralized nuclei in soleus muscles isolated from mice were assessed under high-powered field (200× magnification; five fields per sample).

### Measurement of cell viability

Cell viability was determined by a colorimetric assay using 3-(4,5-dimethyl thiazol-2-yl)- 2,5-diphenyl tetrazolium bromide (MTT) (Sigma). In brief, cells were seeded at 10^4^ cells/well in 96-well plates and incubated in culture medium overnight. The cells were then switched to DM and treated with varying doses of As_2_O_3_ and observed after 96 hr. This assay measures the activity of living cells via mitochondrial dehydrogenase activity that reduces MTT to purple formazan. The formazan was solubilized by DMSO, and its absorbance at 570 nm was measured.

### Statistics

Data are expressed as mean ± SE. We assessed the significant difference from the respective controls for each experimental test condition using analysis of variance and the Bonferroni *t-*test, with *p* < 0.05 considered significant.

## Results

### Arsenic inhibits myogenic differentiation in myoblastic cells

**Figure 1:**
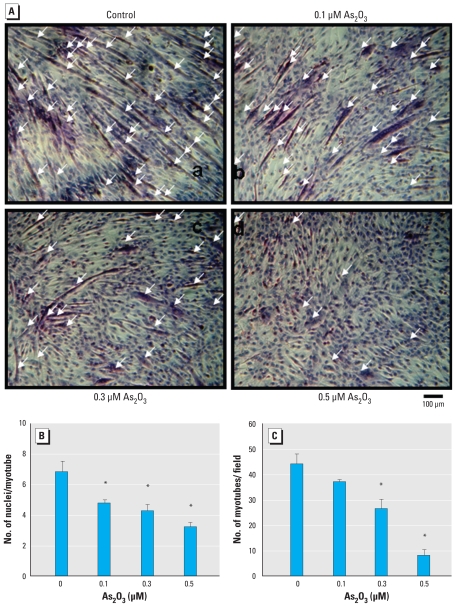
Inhibitory effect of As_2_O_3_ on myogenic differentiation in C2C12 cells; cells were cultured in differentiation medium in the presence or absence of As_2_O_3_ (0.1, 0.3, or 0.5 μM). (*A*) Cell morphology examined by H&E staining at 96 hr; arrows indicate multinucleated myotube formations. (*B*) Number of nuclei per myotube. (*C*) Number of myotubes formed per field, calculated from three random fields per treatment. Data are mean ± SE of three independent experiments. *p < 0.05 compared with control.

**Figure 2:**
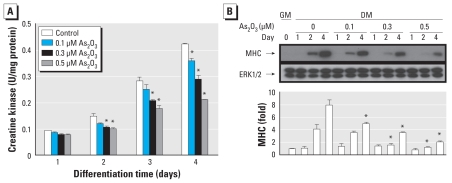
Inhibitory effect of As_2_O_3_ on creatine kinase activity and expression of MHC proteins during myogenic differentiation in C2C12 myoblasts; cells were cultured in growth medium (GM; day 0) or differentiation medium (DM) in the presence or absence of As_2_O_3_ (0.1, 0.3, or 0.5 μM) for 1, 2, or 4 days. (*A*) Activity of creatine kinase. (*B*) Expression of MHC proteins, examined by Western blotting; ERK was used as the loading control. Expression of MHC proteins was quantified by densitometric analysis. Data are mean ± SE for four independent experiments. *p < 0.05 compared with arsenic-unexposed DM control.

We assessed the myogenic differentiation of C2C12 myoblastic cells cultured in differentiation medium morphologically by the alignment, elongation, and fusion of mononucleated myoblasts into multinucleated myotubes. After 4 days, we examined myotube formation by H&E staining (Figure 1A), nuclei per myotube (Figure 1B), number of myotubes per field (Figure 1C), and creatine kinase activity, a marker of myoblast differentiation (Figure 2A). As_2_O_3_ (0.1–0.5 μM) significantly inhibited myotube formation in a dose-dependent manner (Figure 1) but did not affect the viability of C2C12 cells during myogenic differentiation (methylthiotetrazole assay: day 4, As_2_O_3_ 0.1, 0.3, and 0.5 μM; 101.6 ± 2.3%, 97.8 ± 4.6%, and 96.1 ± 3.9% of control, respectively).

**Figure 3:**
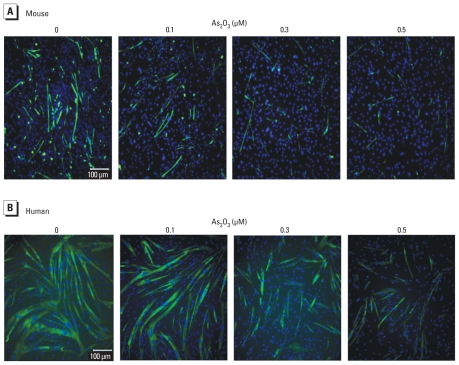
Inhibitory effect of As_2_O_3_ on the expression of MHC proteins in primary mouse (*A*) and human (*B*) myoblasts during myogenic differentiation shown in merged MHC/DAPI images. Results are representative of three independent experiments.

**Figure 4:**
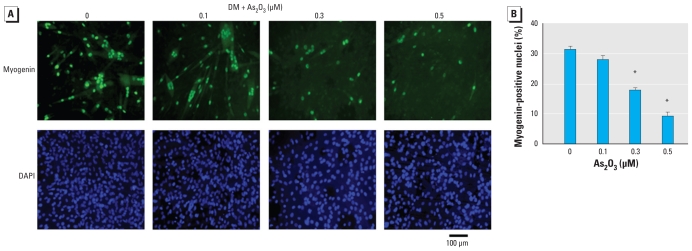
Inhibitory effect of As_2_O_3_ on the expression of myogenin proteins during myogenic differentiation in C2C12 cells; cells were cultured in differentiation medium (DM) for 4 days with or without As_2_O_3_ treatment (0.1–0.5 μM). (*A*) Myogenin expression examined by immunocytochemistry: Top, myogenin expression (green fluorescence); bottom, nuclei stained with DAPI (blue fluorescence). (*B*) Quantification of myogenin expression on day 4 during myogenic differentiation, showing the fraction of myogenin-positive nuclei. Data are mean ± SE of four independent experiments; at least 500 cells in each group were counted. *p < 0.05 compared with control.

**Figure 5:**
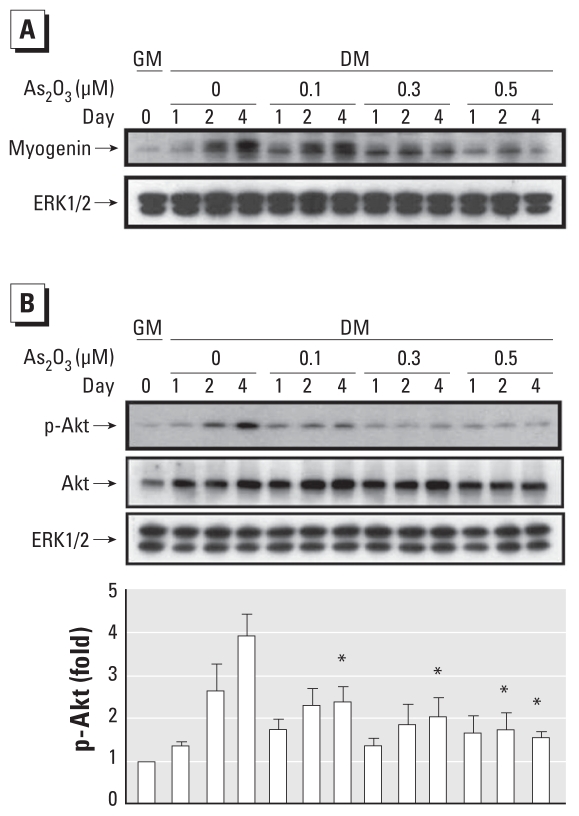
Inhibitory effect of As_2_O_3_ on myogenin protein expression and phosphorylation of Akt protein during myogenic differentiation in C2C12 cells; cells were cultured in growth medium (GM; day 0) or differentiation medium (DM) for 4 days with or without treatment with As_2_O_3_ (0.1–0.5 μM). (*A*) Myogenin protein expression during myogenic differentiation (days 1, 2, and 4) analyzed by Western blotting; ERK was used as the loading control. Results are representative of three independent experiments. (*B*) Phosphorylation of Akt protein examined by Western blotting (top) and quantification of protein expression by densitometric analysis (bottom); results represent the fold increase in proteins relative to the untreated group in GM after normalization to the loading control (ERK1/2). Data are mean ± SE of four independent experiments. *p < 0.05 compared with corresponding arsenic-unexposed controls.

To further characterize the inhibitory effect of As_2_O_3_ on myogenic differentiation, we examined the effect of As_2_O_3_ on the expression of MHC, a late biochemical marker of myogenesis, during myogenic differentiation. MHC expression was detected in C2C12 myoblasts (Figure 2B), primary mouse myoblasts (Figure 3A), and human myoblasts (Figure 3B) during myogenic differentiation. As_2_O_3_ (0.1–0.5 μM) suppressed the expression of MHC in these myoblastic cells. Moreover, myogenin has been shown to induce the expression of the muscle structural gene MHC in *Xenopus laevis* (Charbonnier et al. 2002). We next investigated the effect of As_2_O_3_ on myogenin expression during myogenic differentiation using immunocytochemistry (Figure 4) and Western blotting (Figure 5A). As_2_O_3_ (0.1–0.5 μM) significantly decreased myogenin expression during myogenic differentiation in a dose-dependent manner (Figures 4, 5A). These results indicated that a submicromolar concentration of As_2_O_3_ is capable of inhibiting myoblast differentiation and myotube formation.

### As_2_O_3_ inhibits phosphorylation of Akt, mTOR (mammalian target of rapamycin), and p70s6k

**Figure 6:**
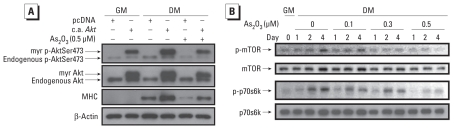
Inhibitory effect of As_2_O_3_ on MHC expression in myoblasts transfected with c.a. *Akt* and the phosphorylation of mTOR and p70s6k proteins during myogenic differentiation. (*A*) Expression of endogenous and mutant proteins in cell lysates analyzed by Western blotting with anti-Akt and phosphorylated Akt (p-AktSer473) antibodies; MHC protein expression was also detected. Equal loading was confirmed by reprobing with anti-β-actin antibody. (*B*) Phosphorylation (p-) of mTOR and p70s6k proteins examined by Western blotting. All results are representative of three independent experiments.

Akt can substitute for PI3K in the stimulation of myogenesis, and it may be an essential downstream component of PI3K-induced muscle differentiation (Jiang et al. 1999). Therefore, we investigated the effects of As_2_O_3_ on the phosphorylation of Akt and its downstream signals, mTOR and p70s6k, during myogenic differentiation. Moreover, Gonzalez et al. (2004) demonstrated that, during myoblast differentiation, Akt kinase activity correlated with Ser473 but not Thr308 phosphorylation. Therefore, we checked Akt (Ser473) phosphorylation during myoblast differentiation. As shown in Figure 5B, Akt protein phosphorylation was gradually activated during myogenic differentiation. As_2_O_3_ (0.1–0.5 μM) significantly decreased the phosphorylation of Akt (Figure 5B) and its downstream targets, mTOR and p70s6k proteins, during myogenic differentiation in a dose-dependent manner Figure 6B). These results imply that As_2_O_3_ inhibits myogenic differentiation by inhibiting an Akt-related pathway.

**Figure 7:**
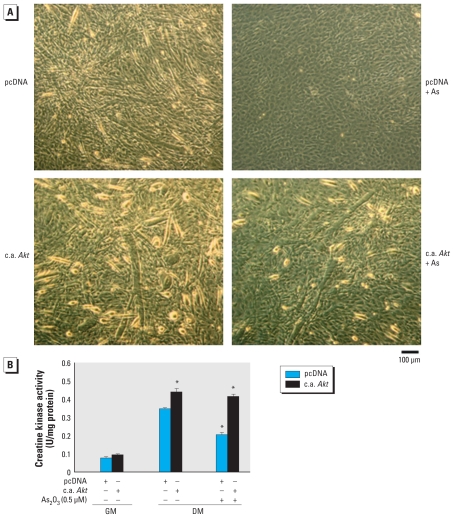
Overexpression of c.a. *Akt* rescues the inhibitory effect of As_2_O_3_ on myogenic differentiation in C2C12 cells transiently transfected with control pcDNA3.1 or c.a. *Akt* in GM for 24 hr. Cells were allowed to differentiate in differentiation medium (DM) in the presence or absence of As_2_O_3_ (0.5 μM) for 4 days. (*A*) Myotubes detected after 4 days in a myogenic differentiation condition; data are representative of at least three independent experiments with similar results. (*B*) Creatine kinase activity; data are mean ± SE for four independent experiments. *p < 0.05 compared with pcDNA control without other drug treatment under DM conditions.

To further confirm the role of Akt signaling in arsenic-induced inhibition of myogenesis, the constitutively active form of *Akt* was overexpressed in C2C12 myoblastic cells. Transfection with a constitutively active form of *Akt* (c.a. *Akt*) enhanced myotube formation (Figure 7A), creatine kinase activity (Figure 7B), and MHC expression (Figure 6A) during myogenesis, and effectively antagonized the myogenesis inhibition induced by 0.5 μM As_2_O_3_ (Figures 6A, 7).

### Arsenic affects skeletal muscle regeneration and function *in vivo*

**Figure 8:**
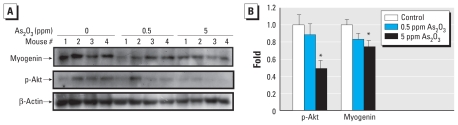
As_2_O_3_ inhibits muscle regeneration after glycerol injury in mice treated with As_2_O_3_ (0.5 and 5 ppm) for 8 weeks. See “Materials and Methods” for details. (*A*) Myogenin expression and Akt phosphorylation in soleus muscles of arsenic-treated mice 3 days after glycerol injection, analyzed by Western blotting. (*B*) Myogenin and phosphorylated Akt proteins quantified by densitometric analysis; results represent protein expression in treated animals relative to untreated controls after normalization to the loading control. Data are mean ± SE for four independent experiments. *p < 0.05 compared with control.

**Figure 9:**
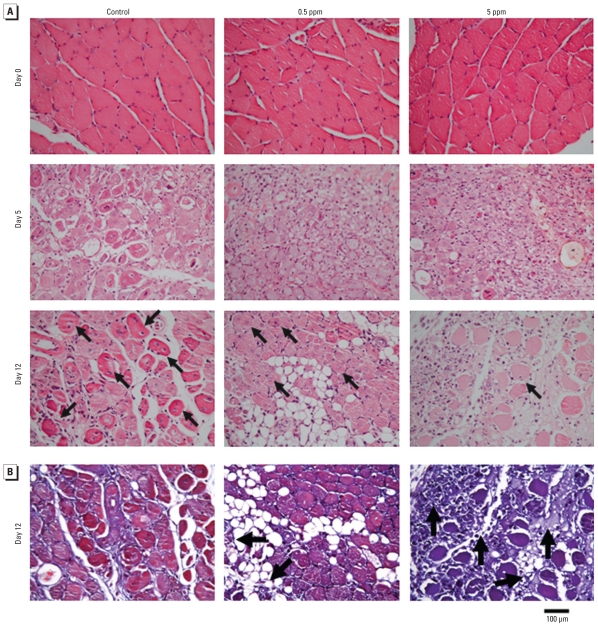
Inhibition of muscle regeneration after glycerol injury in mice treated with As_2_O_3_ (0.5 and 5 ppm) for 8 weeks. See “Materials and Methods” for details. (*A*) Muscle morphology of soleus sections (H&E) before and 5 or 12 days after glycerol injection; centrally localized nuclei indicate myofiber regeneration (arrows). (*B*) Masson’s trichrome staining for collagen (collagen, blue; myofibers, red; nuclei, black). Collagen (fibrosis marker) was distributed around the fat deposits in the injured muscles; note the dense blue-stained fibrous tissue in the As_2_O_3_ (0.5 and 5 ppm) groups (arrows). Results are representative of three independent experiments.

Muscle regeneration in soleus muscle was induced following glycerol injection, which causes extensive and reproducible muscle necrotic injury. The myogenic differentiation is initiated within 2 days followed by extensive regenerative changes within 7–14 days after glycerol injection (Kawai et al. 1990). Glycerol was injected into soleus muscles of control and As_2_O_3_-treated mice (0.5 and 5 ppm As_2_O_3_ in drinking water for 8 weeks). We observed an increase of myogenin expression and Akt phosphorylation in the soleus muscles on day 3 after glycerol injection, but it was suppressed in As_2_O_3_-treated mice compared with control mice (Figure 8). Moreover, H&E-stained cross sections of soleus muscles showed the typical signs of injury and regeneration on day 5 after glycerol injection, as indicated by chronic inflammation (infiltration of mononucleated cells) and newly formed myofibers with centralized nuclei, as well as nearly complete regeneration on day 12, as indicated by the largely restored muscle architecture (Figure 9A). Soleus muscles isolated from As_2_O_3_-treated mice displayed a reduction in myofibers containing centralized nuclei [myofiber regeneration; number (mean ± SE) of regenerating myofibers per high-powered field: control, 52.25 ± 3.57; 0.5 ppm As_2_O_3_,41.50 ± 6.33; 5 ppm As_2_O_3_, 17.25 ± 10.63; *p* < 0.05 vs. control; *n* = 4). In Masson’s trichrome-stained sections on day 12 after glycerol injection, collagen (fibrosis marker) was distributed mainly around the fat deposits in the injured muscles (Figure 9B). The collagen-stained area (fibrosis formation) in soleus muscles isolated from As_2_O_3_-treated mice were obviously larger than those in control soleus muscles (Figure 9B).

## Discussion

Low-dose As_2_O_3_ (0.25 and 0.5 μM) has been reported to activate the transcription of genes involved in adipose differentiation (Salazard et al. 2004). In contrast, Wang et al. (2005) reported that As_2_O_3_ (3 μM) did not induce apoptosis but inhibited differentiation of preadipocyte 3T3-L1 cells. As_2_O_3_ also has been reported to exert dose-dependent dual effects on acute promyelocytic leukemia cells, inducing apoptosis at relatively high concentrations (0.5–2 μM) and inducing partial differentiation at low concentrations (0.1–0.5 μM) (Chen et al. 1997). In addition, high concentrations of As_2_O_3_ (30, 60, and 90 μM) for various periods (24, 48, and 72 hr) caused apoptosis in cardiomyocytes in a dose- and duration-dependent manner (Raghu and Cherian 2009). However, the precise action of inorganic arsenic on skeletal muscle differentiation remains unclear. In the present study, we first demonstrated that low-dose As_2_O_3_ (0.1–0.5 μM) dose-dependently inhibited *in vitro* skeletal muscle differentiation as assessed by myogenin and MHC expression, creatine kinase activity, and formation of multinucleated myotubes without apparent effects on cell viability. Kawai et al. (1990) showed that the extensive regenerative changes in glycerol-induced experimental myopathy, which we observed at 7–14 days after glycerol injection, are similar to those seen in muscles from patients with Duchenne muscular dystrophy. In the present study, using morphologic examination, we also found that As_2_O_3_ (0.5 and 5 ppm) effectively suppressed myogenin expression and retarded muscle regeneration in mouse soleus muscles after injury by glycerol. These results suggest that As_2_O_3_ is capable of inhibiting skeletal muscle differentiation and retarding muscle regeneration.

PI3K and Akt are important signaling molecules in the regulation of proliferation and differentiation in various kinds of cells (Dummler and Hemmings 2007; Rivard 2009). The Akt-related pathway has been shown to be involved in As_2_O_3_-induced inhibition of 3T3-L1 preadipocyte differentiation (Wang et al. 2005). In a recent report, Sandoval et al. (2007) indicated that sodium arsenite triggered the increment or decrement of p53 binding to DNA to affect proliferation or differentiation in epithelial cells through an Akt-related signaling pathway. Previous studies demonstrated an important role of PI3K signaling in myogenesis during an early step of terminal differentiation of skeletal muscle cells (Kaliman et al. 1996, 1998). Jiang et al. (1999) further indicated that serine/threonine protein kinase Akt, a downstream target of PI3K, can substitute for PI3K in the stimulation of myogenesis. Those authors reported that transfection of myr *Akt* in chicken embryo fibroblasts dramatically enhanced myotube formation and expression of the muscle-specific proteins MyoD, creatine kinase, MHC, and desmin. In contrast, transfection of the dominant-negative form of *Akt* decreased the expression of muscle-specific proteins and myotube formation, suggesting that Akt may be an essential downstream component of PI3K-regulated skeletal muscle differentiation. Reduced Akt activation was also associated with inhibition of C2C12 myoblast differentiation (Sumitani et al. 2002). Peng et al. (2003) reported that dwarfism, impaired skin development, skeletal muscle atrophy, delayed bone development, and impeded adipogenesis occurred in mice lacking *Akt1* and *Akt2*. Moreover, activation of Akt in response to insulin stimulation has been shown to involve the phosphorylation of Ser473 and Thr308 residues (Toker and Newton 2000). Paul et al. (2007) also reported that 4-hr exposures to 50 μM arsenite or 2 μM methylarsonous acid inhibited insulin-dependent phosphorylation of Akt on both Ser473 and Thr308 residues in 3T3-L1 adipocytes. However, Gonzalez et al. (2004) demonstrated a specific increase in Akt2 protein levels during myoblast differentiation in which Akt kinase activity was correlated with phosphorylation of Ser473 but not Thr308. Furthermore, Bodine et al. (2001) suggested that Akt-activated mTOR signaling and its downstream targets p70s6k and PHAS-1/4E-BP1 are the crucial regulators of skeletal muscle hypertrophy and that Akt/mTOR activation can prevent skeletal muscle atrophy induced by disuse. In the present study, we found that a submicromolar concentration of As_2_O_3_ significantly and dose-dependently decreased phosphorylation of Akt protein and its downstream targets, mTOR and p70s6k, during myogenic differentiation. The Akt (Ser473) phosphorylation was suppressed by As_2_O_3_ during myoblast differentiation. Overexpression of c.a. *Akt* enhanced myotube formation, creatine kinase activity, and MHC expression during myogenesis and antagonized As_2_O_3_-induced myogenesis inhibition.

Skeletal muscle regeneration is a known adaptive response to muscle injury that occurs through a process of myofiber degeneration, inflammation, and new myofiber formation resulting from satellite cell proliferation and differentiation (Hurme and Kalimo 1992; Kawai et al. 1990). However, fibrosis that also develops during the healing process may hinder muscle regeneration (Chan et al. 2003; Kasemkijwattana et al. 1998). In the present study, we used an experimental glycerol myopathy model to investigate the effect of As_2_O_3_ on skeletal muscle injury and regeneration. Intramuscular injection of 50% glycerol caused extensive muscle fiber necrosis and loss of muscle mass, which requires myogenesis during the initial phase of recovery to restore muscle mass (Kawai et al. 1990). Our results indicate that As_2_O_3_ in drinking water impaired muscle regeneration and increased fibrosis (based on increased collagen staining) in a murine model of glycerol injury. Moreover, the increase in Akt phosphorylation observed 3 days after glycerol injection in the soleus muscles of control animals was markedly suppressed in animals treated with As_2_O_3_. Therefore, these results suggest that As_2_O_3_ inhibited myogenic differentiation by inhibiting an Akt-dependent pathway.

## Conclusions

Akt is an important signaling pathway involved in myogenesis. Here, we found that a submicromolar concentration of As_2_O_3_ significantly and dose-dependently inhibited myogenic differentiation by inhibiting an Akt signaling pathway. Moreover, As_2_O_3_ suppressed skeletal muscle differentiation and regeneration after injury in an *in vivo* mouse skeletal muscle regeneration model. Taken as a whole, these *in vitro* and *in vivo* findings suggest that As_2_O_3_ may be an environmental risk factor for myogenesis.
